# After-school sports programmes and social inclusion processes in culturally diverse contexts: Results of an international multicase study

**DOI:** 10.3389/fpsyg.2023.1122362

**Published:** 2023-03-22

**Authors:** Bastian Carter-Thuillier, Víctor López-Pastor, Francisco Gallardo-Fuentes, Juan Carter-Beltran, Juan-Miguel Fernández-Balboa, Pedro Delgado-Floody, Elke Grimminger-Seidensticker, Andrew Sortwell

**Affiliations:** ^1^Department of Education, Universidad de Los Lagos, Osorno, Chile; ^2^Programa de Investigación en Deporte, Sociedad y Buen Vivir, Universidad de Los Lagos, Osorno, Chile; ^3^Facultad de Educación, Universidad Católica de Temuco, Temuco, Chile; ^4^Facultad de Educación, Universidad de Valladolid, Segovia, Spain; ^5^Department of Physical Activity Sciences, Universidad de Los Lagos, Osorno, Chile; ^6^Facultad de Formación de Profesorado y Educación, Universidad Autónoma de Madrid, Madrid, Spain; ^7^Department of Physical Education, Sports and Recreation, Universidad de La Frontera, Temuco, Chile; ^8^Department of Physical Education and Sports, Faculty of Sports Science, University of Granada, Granada, Spain; ^9^Department of Exercise and Health, Paderborn University, Paderborn, Germany; ^10^School of Nursing, Midwifery, Health Sciences and Physiotherapy, University of Notre Dame Australia, Sydney, NSW, Australia; ^11^School of Education, Excelsia College, Sydney, NSW, Australia

**Keywords:** social inclusion, immigrant students, Mapuche, after-school programmes, sports programmes, ethnic groups

## Abstract

This research aimed to understand the role of after-school sports programs in social inclusion processes in culturally diverse contexts through a multicase study within two locations. The first location was in Spain where immigrant and Spanish students were enrolled, and the other was in Chile with Mapuche-Huilliche students, immigrant and Chilean students. The implemented programs at both sites were similar in their educational focus on socio-educational values, and teaching models (hybridization of teaching games for understanding and cooperative learning) that enhance social inclusion. Using individual and group interviews with teachers, sports coordinators, parents, and students, a qualitative approach was used to identify the factors that facilitate or hinder the social inclusion processes. In addition, the researchers used qualitative observations of the programs over six months using “notes logbook” to record their impressions during the observation process. Results indicated that the implemented sports programs successfully facilitated social inclusion processes, enabling the development of interpersonal skills and relationships between students from different cultural backgrounds. The previous training and experiences of teachers in culturally diverse contexts, and incorporation of traditional sporting games from all cultures, seems to be an important facilitator factor for the inclusion potential of the implemented programs.

## 1. Introduction

The current reality of a globalized world generates the intensification of cultural diversity in society (Civitillo et al., 2017), transforming school contexts through the social fabric change and new relationships building in the classrooms (Fruja, 2019). This increase in a more culturally diverse classroom environment has positioned the country’s educational school settings as transcendental spaces for the early promotion of intercultural skills (Zhang and Zhou, 2019), by being challenged to recognize different cultural identities and ethnic groups under a framework of social equity (Graham, 2016). School-based sports programs are recognized as influencing and shaping students’ acquisition of values, self-management and interpersonal skills, along with movement skill competencies that contribute to their cognitive, emotional, social and motor development and facilitate inclusion in society, whilst also preparing them for the future (Bessa et al., 2019).

In schools across Europe, Latin America, and Australia, cultural diversity is reflected through multiple expressions and identities (Wiium and Dimitrova, 2019). For example, in Spain and Chile, immigration has been a factor in recent years that has contributed significantly to the increase in the cultural diversity of schools (Berríos-Riquelme et al., 2021; Martínez-Rojas et al., 2021). In Chile, members of indigenous peoples (e.g., Mapuche as a macroethnic group composed of four collectives: Mapuche-Picunche, Mapuche-Huilliche, Mapuche-Pehuenche, and Mapuche-Lafquenche) also have a high presence in the school system, representing specific cultures, worldviews, and ancestral knowledge (MDSF, 2018; De la Maza and Bolomey, 2019).

Currently in Spain, the percentage of the foreign population is up to 11.5% (INE, 2022), while in Chile, it recently is at its highest historical percentage of 7.5% (INE, 2021). These percentages of the foreign population are reflected in schools in both countries (Estalayo et al., 2021; Carter-Thuillier et al., 2022). In Spain, 11.6% of the students come from countries other than Spain (Fundación Europea Sociedad y Educación, 2020), while in Chile, the enrollment of immigrant students has quadrupled during the period 2014–2018, currently reaching 5.3% (SJM, 2022). Likewise, in the latter country, members of indigenous peoples have historically represented a high percentage of the population. In fact, according to the latest official statistics, the Mapuche people (composed of the previously named four collectives) are the main ethnic group which represents 9.9% of the national population (CASEN, 2017) and 2.5% of all school students of the country (MDSF, 2016). In short, cultural diversity is a relevant challenge for schools in Spain and Chile.

In each context, unique relationships are shaped between members of the migrant population, different ethnic groups and the rest of society (Banks, 2016). However, different studies have shown that in both countries, immigrant students are constantly at risk of segregation and social exclusion, especially when they are from a low socio-economic family background and had a precarious schooling situation in their original countries (Goenechea, 2016; Lara, 2017; Caqueo-Urízar et al., 2021; Martínez-Rojas et al., 2021; Martinez, 2022). Likewise, recent research indicates that in Chile and Spain, schools have usually been the setting where local cultures are taught, and often imposed upon immigrant students leading to them relinquishing their own culture and assimilating into the culture of the receiving country (García-Yepes, 2017; Pincheira, 2021). This has sometimes led to resistance and rejection by immigrant students in both contexts (Lara, 2017; Carrillo-Sánchez, 2021). The situation of Chilean indigenous peoples is historically similar, as the school has systematically imposed the hegemonic culture through a monocultural curriculum that does not provide space for the customs, beliefs and ancestral knowledge of the ethnic groups (Breidlid and Botha, 2015; Turra et al., 2017). Specifically, Mapuche ethnic groups for decades have suffered a significant weakening of cultural identity through school education, consistently forcing them to deny their linguistic, bodily and educative practices (Carter-Thuillier et al., 2018; Caqueo-Urízar et al., 2021). These assimilation processes are favored by discourses that define cultural diversity as having a negative impact on educational and social aims (Hellgren and Gabrielli, 2021; Salas et al., 2021; Webb, 2021; Vollrath, 2022), and legitimize isolation and exclusion strategies toward students belonging to immigrant or indigenous groups (Plenty and Jonsson, 2017). However, according to Berry (2005) imposed assimilation from the dominant culture usually generates reactive behaviors in minority groups, which is the case of the Mapuche-Huilliches, who, for decades, have systematically opposed the policies of the Chilean State (Zuñiga and Olate, 2017).

Finally, the above-described situation has generated the need to design and implement educational strategies based on dialogue and respect among different ethnic groups in order to facilitate social inclusion (Carter-Thuillier et al., 2022) *via* positive experiences within culturally diverse contexts (Juvonen et al., 2018; Smith et al., 2019; Lleixà and Nieva, 2020). In this regard, sport has shown a clear potential for social inclusion in migration and ethnic contexts (Künis et al., 2016; Burrmann et al., 2017; Smith et al., 2019; Middleton et al., 2020), since it is a supra-cultural (Paredes and Reina, 2006) and transcultural (Heinemann, 2022) space that can serve as a meeting point for positive interaction (Ekholm, 2018; Middleton et al., 2020; Mohammadi, 2022). In fact, various scholars (Skille and Fahlén, 2020; Papageorgiou et al., 2021; Pedersen et al., 2021) have argued that sports activities can be a favorable space for the development positive social interactions between people from different cultures, especially when developed using an “intercultural” approach, which not only implies eliminating fear and prejudice toward “others,” but also assuming interaction as an opportunity for learning and communication. Thus, the universal framework offered by motor praxeology as a possibility of human action can facilitate the development of social interactions in culturally diverse contexts (Bortolotti, 2021). Furthermore, the particular characteristics of sport make its application very viable and useful in cultural diversity school-contexts to promote social inclusion without losing cultural identities (Cockburn, 2017; Flensner et al., 2021). However, sports can also spark conflicts, foster racial stereotypes, and perpetuate unbridled rivalries among ethnic groups, cultures, and nationalities (Barker et al., 2013; Van Sterkenburg et al., 2019; Kavanagh, 2022). Therefore, it needs to be carefully planned, examined, and implemented to become a true alternative to these collateral risks (Grimminger-Seidensticker and Möhwald, 2017). Especially, the educators’ intercultural competence or sensitivity toward cultural diversity might play a crucial role in implementing intercultural education programs in sports (Grimminger, 2012).

As such, in this article we will refer to the term “school sports” (SCSP) as all sport activities that are practiced at school for educational purposes, out of physical education class but usually in after-school programs, focusing on the development of personal and social values as well as on physical performance, in coherence with the school curriculum (Blázquez, 2010). Among such values, issues of cultural identity, cultural diversity, and social inclusion are listed to enhance reciprocal knowledge of the customs, practices and world views of all students in combination with the promotion of motor skills (Jiménez-Herranz et al., 2016; Carter-Thuillier et al., 2018; Fernández-Gavira et al., 2018). However, there is rarely research about the effect of such SCSP programs on the aspired pedagogical aims. The few systematic reviews (Fernández et al., 2013; Carter-Thuillier et al., 2017; Heath et al., 2018; Shields and Lujan, 2018) of the last decade have shown that there are few reports on the effects of SCSP programs in contexts of immigration and ethnic groups, or whether these initiatives developed in school settings manage to use the potential of sport to really favor the preservation of identities and the building of positive intercultural relations.

Specifically, the work of Fernández et al. (2013) shows that most of the research on foreign population and sport concentrates on “migration of sport talents” and “sociological studies,” with school being a context where practically no research is carried out in this regard. In the case of Carter-Thuillier et al. (2017), it is noted that over the last 5 years the volume of research on the use of sport in schools to promote social inclusion processes where there are people of different nationalities and cultures has slowly grown. Shields and Lujan (2018) review exhibit how participation in sporting activities contributes to social inclusion in contexts with a migrant population, while Heath et al. (2018) show how participation in extracurricular activities (e.g., SCSP) fosters the socialization of young migrant populations.

## 2. Purpose of the study

To explore the role of SCSP programs on social inclusion processes in culturally diverse contexts, in Spain and Chile. The first context for the SCSP program in Spain involved immigrant and Spanish enrolled students. The second context is a SCSP program in Chile with Mapuche-Huilliche students (Chilean nationality), immigrant students and Chilean students (non-members of indigenous groups).

## 3. Materials and methods

A qualitative multi-case study was conducted. In particular an ethnography of two case studies was carried out with two specific groups, each composed of individuals with similar and shared traits, habits, and contexts. This double source of data was useful to understand the commonalities and differences of both cases (Stake, 1995). As such, qualitative techniques and instruments were used to identify and analyze traits stemming from participants’ perceptions, ideas, and motivations regarding the process of social inclusion through SCSP.

### 3.1. Contexts and participants

The first case study aimed to analyze the social inclusion processes in a SCSP program in Spain and focused on the interactions [according to American Psychological Association [APA]., 2022), any process that involves reciprocal stimulation or response between two or more individuals] between migrant students from different nationalities and their relationships with other actors in the context: classmates of different nationalities, teachers, program coordinators, family members, among others. In this study, only those students with a nationality other than Spanish have been considered immigrant students.

The Spanish case study was part of a larger research project developed jointly by a City Council and a University. It aimed at developing a SCSP program with social transformation purposes to intervene upon and modify certain negative dynamics that had emerged in previous sporting contexts during the school stage between native and migrant students, while focusing on promoting social and civic values. Given its formative and inclusive approach, not use a competitive logic and early sport specialization, this SCSP program had features that make its analysis useful and valuable, e.g., promotion of values such as cooperation, solidarity, mutual respect, etc.). This SCSP program was implemented during the school year in 24 school centers. It took place after school, every week, Monday and Thursday, with 2-h training sessions. On Fridays, sporting meetings were held for students from all schools. On that day, broader meets were carried out, with a high number of students, since the teams were composed of students from different educational centers. For that purpose, sports activities of short duration were organized related to the sport being studied in the general school programming. No official results or standings were registered.

All class sessions were designed based on a hybrid model that mixed “Teaching Games for Understanding” (TGfU) and a cooperative learning approach. The TGfU was implemented considering the principles proposed by Tan et al. (2012): (a) sampling; (b) tactical complexity; (c) representation; and (d) exaggeration. The cooperative learning approach followed the recommendations of Dysob and Casey (2016): (a) positive interdependence; (b) interpersonal and small groups skills; (c) face-to-face interaction; (d) individual accountability; and (e) group processing. Both models have pedagogical characteristics and structural principles that allow their complementary use (Fernandez-Rio, 2014), this means: (a) a student-centered process; (b) learning in participative contexts; (c) positive interdependence; (d) enhanced responsibility among students; (e) authentic learning activities; (f) focusing on social, physical, and cognitive development processes; and (g) active learning through decision-making, social interaction, and cognitive comprehension. Pedagogical principles outlined above (hybrid model of TGfU and Cooperative Learning) were displayed with the following sports activities: (a) basketball; (b) handball; (c) futsal; (d) track and field; (e) volleyball; and (f) rugby. Additionally, it worked all year: (a) activities to learn to run at aerobic rhythms; (b) social values and living habits; (c) basic aspects to practice physical activity (warm-up, heart rate control, hydration, etc.); and (d) traditional sporting games (TSG) related to the cultures present in the classroom. Some examples of TSG observed in the sessions: “Chirra” a TSG from North Africa similar to hockey, the “seven stones” a typical collective TSG of Central America and the Caribbean where a “tower” of balls is built and teams try to knock it down by throwing small balls from a distance of approximately 7 m, “Blind Hen” TSG from of South American countries where a child blindfolds and tries to catch the rest of his classmates, among others. For more details on these games, see Bantulá and Mora (2005).

The observed participants were 114 students (56 immigrants: 34 boys and 22 girls),with range age 7–12 years (*M* = 9.43; SD = 1.56). They belonged to 11 different groups of SCSP, operating in six schools (the schools with the highest percentage of immigrant students were selected). All migrant students were first generation in the country and all of them were born abroad, and came mostly from North Africa, Eastern Europe, East Asia, South America, Central America, and the Caribbean. Spanish students (35 boys and 23 girls) were of the same ages as their migrant counterparts. All this information was provided by the program coordination.

The study also involved eight teachers, who implemented the SCSP program. Some of them were pre-service teachers in the last year of their university education. their ages ranged between 21 and 30 years (*M* = 23.87; SD = 2.71). To work in the SCSP program, teachers had to pass a specific training course, focusing on: methodology, session structure, organization and control of the group, warm-up, conventional sports and alternative sports, physical-sports activity and healthy habits, education in values, and conflict resolution. They also learned basic principles of intercultural education and TSG, subjects were taught by the program coordination and professionals from the university. Specifically, with regard to interculturality, teachers received training on values associated with respect and attention to cultural diversity, the positive recognition of cultures and strategies to promote interaction between people from different cultures. In regards to the training on TSG, traditional games from the countries present in the SCSP program were shared and explained since incorporating games from different cultures into the classroom is a favorable tool for recognizing and strengthening different cultural identities, and can also have a positive impact on the emotional state of students, their motor development, their ability to take decisions and the development of coexistence based on mutual respect (Pic et al., 2019; Martín-Martínez et al., 2021; Lavega-Burgués et al., 2023; Moya-Higueras et al., 2023). The program coordinator (male, 36 years old), who also participated in the study and had a specific training in physical education. His task was to supervise the teachers’ work and to ensure that all SCSP groups function properly on an educational and organizational level.

The second case study aimed at analyzing the social inclusion processes in an SCSP program at a south macrozone city of Chile, focused on the interactions of Mapuche-Huilliche students, immigrant students and their relationships with others agents who participates in the context. The schools provided information on who are Mapuche and immigrant students. In this sense, students are recognized as Mapuche-Huilliche if they are enrolled in an indigenous community of the Chilean state and also recognize themselves as members of this people, in addition to having one (at least) last name that links them to this group. A total of 41.6% of them live in indigenous communities with other Mapuche-Huilliche people. In the case of immigrant students, they are those who have a nationality other than Chilean and are all foreign-born. The implementation of the SCSP program in Chile was legitimated by existing racist and discriminatory dynamics, particularly against Mapuche-Huilliches and migrants, as also reported in the introduction.

In this case study, 46 students (28 boys and 18 girls) were observed (24 of which were Mapuche-Huilliche students, 15 boys and 9 girls; 4 immigrants from Colombia and Venezuela, 2 boys and 2 girls), with ages ranging 7–12 years (*M* = 9.50; SD = 1.84). These participants belonged to two schools (four SCSP groups). This study also involved six parents or legal guardians of Mapuche-Huilliche and migrant students who were invited through the schools and agreed to participate voluntarily.

This program executed at Chile was created with analogous characteristics of the Spanish SCSP program, using in all groups the same education principles (focus on socio-educational values), teaching models (combining TGfU and cooperative learning) with the aim of enhancing social inclusion processes. The program followed also the same organizational structure: two school SCSP sessions were held each week, and on Fridays, formal sports meetings (all SCSP students groups came) took place in the centers. The pedagogical principles displayed in game situations are the same as in the first case study, including some playful-physical practices from the Mapuche culture (e.g., Mapuche ethnic TGS like Palin or Linao) (Poblete et al., 2020). Moreover, cooperative learning activities and situations were implemented into the program, to foster positive social relationships among all participants.

In addition to the students, two male teachers (21–30 years old; *M* = 25.5; SD = 4.5) and a male coordinator (30 years old) participated in the study; they all were specialists in Physical Education. Teachers’ role was to implement the learning activities in each SCSP group; therefore, they received specific training on the same topics as their Spanish counterparts, the program coordinator and university professors were in charge of this training.

### 3.2. Data collection

The data collection techniques and instruments used in both case studies were: (a) participatory observation (6 months in each context), as, according to DeWalt and DeWalt (2011), it allows for systematic collection in naturalistic settings (e.g., communities of many different cultures) to understand the fundamental processes and patterns of these social spaces, (b) 18 in-depth individual interviews of key informants, and (c) 12 focus-group interviews with students. Sangaramoorthy and Kroeger (2020) state that individual in-depth interviews and focus groups can complement each other as data collection techniques, and can help to deepen the findings of systematic observations during ethnographic processes. In addition, the researchers used a “logbook,” whereby they noted comments and preliminary conclusions of the research process (Whaley, 2014).

Following the suggestion by Taylor et al. (2015), observations non-structured was developed focusing on the interactions of the students (immigrants in Spain, Mapuche-Huilliche and immigrants in Chile) with the other actors. For the interviews, as recommended by Taylor and Bogdan (2000), no closed questions were defined, but a list of topics was used to guide the conversation focused on social interactions of actors and social inclusion processes. Some examples: (i) interpersonal relations between immigrant, and Mapuche-Huilliche in Chilean case, students who participate in SCPS programs; (ii) interpersonal relations of immigrant students, and Mapuche-Huilliche in the case of Chile, with students who do not belong to cultural minorities; (iii) pedagogical practices that foster positive intercultural relations in the classroom; (iv) relationship between teacher and students from cultural minorities; (v) culturally motivated conflicts in the classroom; and (vi) conflicts occurring in the classroom due to cultural causes; among other issues. As for the individual in-depth interviews with key informants, in the two case study nine were undertaken (Spain: eight with teachers and one with coordinator; Chile: two with teachers; one with coordinator, and six with parents or legal guardians). Furthermore, while eight focus-group interviews were organized with immigrant students in the Spanish study (the students were subdivided into: eight groups of seven members), four group interviews took place in the Chilean context with students belonging to the Mapuche-Huilliche ethnic group and migrant students (here, the students were subdivided into: four groups of seven members).

### 3.3. Data analysis

Interviews and observations were full transcribed. The technique of “content analysis” (Taylor et al., 2015) was used to select, organize, and classify the data. This type of analysis allowed the researchers to formulate reproducible and valid inferences for each context studied. The process consisted of three phases: (a) discovery; (b) coding; and (c) relativizing of the data. Table 1 shows the categories and subcategories that emerged from the analysis used for both case studies.

**TABLE 1:** Emerged categories and subcategories of analysis.

Categories	Subcategories
Interpersonal relationships between students of cultural minority groups.	Relationships between immigrant students in Spanish context
	Relationships between Mapuche-Huilliche and immigrant students in Chilean context.
Interpersonal relationships between cultural minorities students and non-cultural minority children.	Relationships between immigrant students with native Spanish children.
	Relationships of immigrant and Mapuche-Huilliche students with Chilean children non-member of cultural minorities groups.
Interpersonal relationships between SCSP teachers and cultural minority students.	Relationships between SCSP teachers and immigrant students in Spanish context.
	Relationships of SCSP teachers with Mapuche-Huilliche and immigrant students in Chilean context.
Tensions generated by cultural stereotypes about the body and the practice of physical sporting activities.	Tensions caused by cultural stereotypes in Spanish context.
	Tensions caused by cultural stereotypes in Chilean context.

### 3.4. Ethics and trustworthiness

The studies involving human participants were reviewed and approved by the Academic Committee of the Doctoral Program in Transdisciplinary Research in Education at University of Valladolid (Spain) and the Bioethics and Biosafety Committee of the Universidad de Los Lagos (Chile). Written informed consent to participate in this study was provided by the participants and their legal guardians as appropriate in each case.

Specifically, In both case studies, four ethical criteria were considered: (a) informed consent of parents or legal guardians, teachers, and coordinators of each program; (b) informed assent of each student; (c) confidentiality of the identity of the participants; and (d) communication of the aims of the research to the participants. No one refused to participate.

In addition, the four trustworthiness criteria recommended by Lincoln et al. (2011) were used: (a) credibility, through systematic observation for 6 months; (b) transferability, through the dense description of the contexts and populations studied; (c) dependence, through triangulation and reciprocal use of techniques and instruments to provide greater neutrality; and (d) confirmability, by exposing the interests of the research team from the beginning and, also, triangulating the sources and instruments.

## 4. Results

**FIGURE 1:**
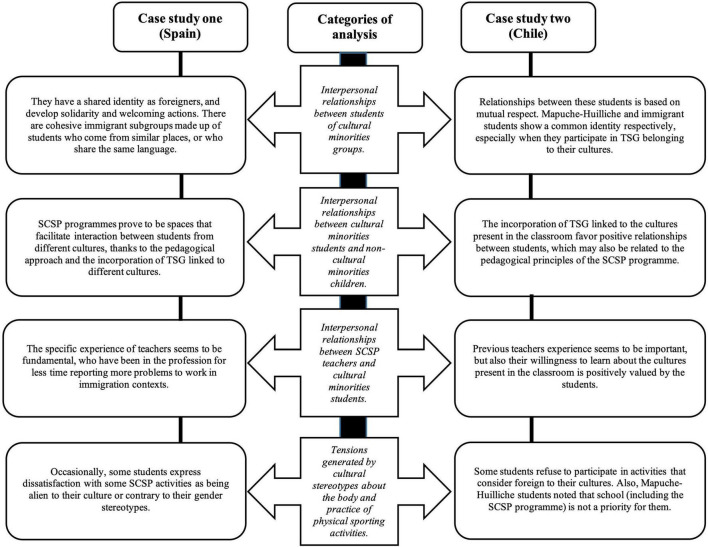
Summary of study results.

The results are divided into four main categories: (a) interpersonal relationships between students of cultural minority groups, (b) interpersonal relationships between cultural minorities students and non-cultural minority children, (c) interpersonal relationships between SCSP teachers and cultural minority students, and (d) tensions generated by cultural stereotypes about the body and the practice of physical sporting activities. Each of these categories has its corresponding sub-categories. Furthermore, some illustrative textual quotations obtained from the observations and interviews are presented in each instance. Table 2 contains the sources of data collection and their respective codes used to identify such sources in the categorization and subcategorization of the data and their corresponding codes of analysis. Also, a summary of the main results for each category can also be seen in Figure 1.

**TABLE 2:** Sources of data collections and their corresponding codes of analysis.

Sources of data collection	Codes
Observation class of SCSP – Spanish context – class number.	O-SP-N
Observation class of SCSP – Chilean context – class number.	O-CH-N
In-depth interview – coordinator SCSP – Spanish context.	DI-C-SP
In-depth interview – coordinator SCSP – Chilean context.	DI-C-CH
In-depth interview – parents – Chilean context – person number.	DI-P-CH-N
In-depth interview – teacher of SCSP – Spanish context – person number.	DI-T-SP-N
In-depth interview – teacher of SCSP – Chilean context – person number.	DI-T-CH-N
Group interview – male immigrant – Spanish context – person number.	GI-MI-SP-N
Group interview – female immigrant – Spanish context – person number	GI-FI-SP-N
Group interview – male immigrant – Chilean context – person number.	GI-MI-CH-N
Group interview – female immigrant – Chilean context – person number.	GI-FI-CH-N
Group interview – male Mapuche-Huilliche – Chilean context – person number.	GI-MM-CH-N
Group interview – female Mapuche-Huilliche – Chilean context – person number.	GI-FM-CH-N

### 4.1. Interpersonal relationships between students of cultural minority groups

This category represents the social interactions of immigrant and Mapuche-Huilliche participants on each case study, specifically: (a) the relations between the immigrant students within the Spanish program; and (b) relations between the Mapuche-Huilliche students and their immigrant companions.

Both case studies, the results show the existence of particular dynamics and types of interplays that stem from the respective cultural identity. In this sense, two subcategories emerged: (a) relationships with immigrant students in Spain and (b) relationships between students belonging to the Mapuche-Huilliche ethnic group in Chile.

#### 4.1.1. Subcategory: Relationships between immigrant students in Spanish context

Although immigrant students come from different nationalities, they all see themselves as outsiders with regard to the main culture. As such, they must endure discriminatory situations that are associated with their common circumstances (e.g., being called “foreigners” or having segregation experiences at school). Curiously enough, this not only promotes a sense of belonging and cohesion, but also encourages the existence of specific codes of communication and identity among these groups.

An example of the aforementioned dynamics is what happens when new immigrants begin to participate in the SCSP program. Those who have more experience in the program develop welcoming and solidarity practices, based on empathy toward their new immigrant peers. These attitudes of collaboration are not observed when new Spanish children arrive, which shows that relations between immigrant students have specific characteristics. Following are two instances:

*We are all from other countries, so we help each other* (GI-MI-SP-03).

* Today a new foreign student arrived. He does not speak Spanish and has difficulties understanding the teacher’s instructions. However, other foreigners helped him, accompanying him in the activities* (O-SP-12).

Furthermore, there are subgroups of immigrants usually made up of students who come from similar places (South America, Central America, North Africa, etc.) or who share a common language. These aspects facilitate communication and a shared sense of identity among these individuals, as well as facilitating cohesion within these sub-groups.

In fact, there are subsets of immigrants who use their native languages to communicate during SCSP sessions for two main reasons: (a) when they want no one else to understand what they say and (b) to take advantage of some sports situation (e.g., when discussing a strategy to score a goal).

*Two students from Morocco speak in Arabic. Apparently, they are making a plan to score a goal, without the rivals knowing how. They have confirmed this assumption of mine when I asked them afterwards* (O-SP-19).

In some SCSP sessions, conflicting situations were generated among immigrant students. In most cases, they were resolved, peacefully, through dialogue; however, sometimes it was observed that some migrant students wanted to resolve conflict situations through physical and/or verbal violence, assuming that these practices are normal and legitimate: *“Today, a just arrived immigrant student got angry when a classmate made a mistake, and pushed and threated to throw a punch at him”* (O-SP20). This, no doubt, negatively conditioned personal relationships. Nonetheless, it was observed that, as the program advanced, these aggressive behaviors diminished and practically stopped at the end of the year.

#### 4.1.2. Subcategory: Relationships between Mapuche-Huilliche and immigrant students in Chilean context

The interpersonal relationships of Mapuche-Huilliche students had singular characteristics that differentiate them from other types of interactions. To a great extent, these can be associated with the cultural particularities and the worldview of this ethnic group-aspects that, logically, transversally affected the relationships developed by these individuals with respect to the rest of the group of students.

Although the Mapuche-Huilliche people have their own language, students hardly use it to communicate. When asked why, they expressed that they cannot speak in their ethnic language because, when they do so, (a) teachers forbid it and, at times, punish them for doing so, and (b) their non-Mapuche classmates reject them. Here are examples of both:

*I do not talk [in Mapuche-Huilliche language] at school* (GI-FM-CH-01).

* Last year, a teacher told me that everybody in school had to speak in Spanish, because it is the language spoken in Chile* (GI-MM-CH-03).

* My classmates will make fun of me [if they hear me speak in Mapuche-Huilliche language]* (GI-MM-CH-06).

However, it can be observed that these students share some discourses and practices that are configured as symbols of a shared identity and provide them with a sense of belonging to the Mapuche-Huilliche culture. Concretely, these students show a high sense of group cohesion when TSG linked to their ethnicity are incorporated into the program. This implies that these activities are configured as elements and identity traits of their culture, showing that these students recognize themselves as part of a particular group: “*Today games of the Mapuche-Huilliche ethnic group are held, the students of that culture show interest in the activity and try to be teammates, expressing that the game belongs to them*” (O-CH-33).

Moreover, the relations between Mapuche-Huilliche students are built mostly on a framework of mutual respect, although there are some conflicts. There is a number of minority students that seek being protagonists by demonstrating their skills in some sports practices. When these students are new to the program, they tend to aggravate their older teammates who might be less skillful in game situations.

The sporadic conflicting episodes among these students tended to decrease gradually as the school year progressed. These situations, however, were not observed among Mapuche-Huilliche students who had been participating in the program for longer. Both situations may be related to the promotion (or lack thereof) of educational values and the use of cooperative learning strategies that promote the positive interdependence of students within the program: “*I used to fight him [referring to another Mapuche-Huilliche student], but now we get along because we play together*” (GI-MM-CH-04).

In this sense, the relationships between immigrant students are mostly based on respect. Despite coming from different countries, they seem to recognize each other as having a common identity as foreigners, as is evident in the following sentence from an immigrant student: “*we became friends, because we are both from another country and we have another culture, we like other games (…) we miss our countries too (…) we are similar*” (GI-FI-CH-02).

Conflicts also develop between immigrant students, especially on those occasions of play where some children perceive themselves as less competent than others. On some minority occasions, they also argue when, as a result of some play action, one classmate makes fun of another.

*We are friends (other foreign peers), but sometimes I get angry when things don’t work out when I play, even more so when my friends laugh at me* (GI-MI-CH-01).

Interactions between Mapuche-Huilliche students and children from other countries are based on respect for differences. There are also practices of mutual support between them, where at times it is observed that they all recognize each other as members of cultural minorities, as a Colombian student points out: “*here everyone should have the right to live their culture* (…) *but in Chile sometimes they discriminate against us, as happens to our classmates who are Mapuche*” GI-MI-CH-03. Although there are also conflicts over play situations, for the same reasons as mentioned above (teasing in play situations or reproaches when a classmate makes a mistake).

### 4.2. Interpersonal relationships between cultural minorities students and non-cultural minority children

This category focuses how social relationships are configured between cultural minorities individuals with other children in both studies and shows how this affects the inclusion processes. Here also are two subcategories: (a) social interactions that immigrant students develop with native Spanish children and (b) relationships between Mapuche-Huilliche and non-Mapuche-Huilliche students.

#### 4.2.1. Subcategory: Relationships between immigrant students with native Spanish children

As mentioned above, immigrant and native students attend Friday sporting events, where students from different schools participate. Here, the relationships between students of foreign origin and Spanish students are, basically, based on mutual respect, despite the fact that both groups hold different worldviews, cultural habits and nationalities: “*In all teams there are children of different nationalities. Spanish and foreign children have played the whole class with no conflict*” (O-SP-26).

One of the reasons for such harmony might be that everyone participates in games for pedagogical purposes, where there is no record of results or classifications. The teams are always composed of boys and girls from different educational centers, aiming to promote equality between them. This is also the case with teams composed of natives and immigrants. The lack of conflict seems to point out the success of this pedagogical methodology, which fosters the acquisition of new social capital, positive social interactions among all, and the creation of networks among the individuals of all groups. It is possible then, that favors the interaction of students with different cultural backgrounds from a pedagogical framework that promotes the development of intercultural values (focusing on respect and positive valuing of cultural diversity) and without to accentuate competitiveness (through the reduction of rivalries, without generating winners and losers), be the key to reduce the risk of conflicts. This is evidenced in this quote: “*I made several Spanish friends on Friday, they are from other schools, very ‘cool.’ They are so funny*” (GI-MI-SP-13).

However, occasionally, there emerged some conflicts accompanied by ethnocentric discourses due to the mutual prejudices between immigrants and natives. For instance, some Spanish students expressed derogatory opinions toward their immigrant peers, assuming that because of their foreign status the latter are intrinsically inferior (especially if they come from poor countries). Oddly enough, this led to a few cases where immigrant students publicly deny their foreign origin so as not to be discriminated against.

*A Spanish student tells another classmate: “these blacks are useless,” regarding a fellow immigrant who made a mistake during the game* (O-SP-08).

* A Colombian child refuses to come from that country several times, does not want to be discriminated against. [He arrived two years ago to Spain]* (O-SP-14).

In turn, there was similar language used by some foreign students against their Spanish peers, generating even more distress and some threats against them regarding the sporting activities or for other reasons. However, in this regard, it must be said that most of these cases were instigated by students who had been in the program for a significantly short time (less than 3 months) and were still unfamiliar with the educational values taught there. Also, it was one case in which a novice teacher whose lack of experience prevented him from anticipating and aborting the conflict.

#### 4.2.2. Subcategory: Relationships of immigrant and Mapuche-Huilliche students with Chilean children non-member of cultural minorities groups

The relations between Mapuche-Huilliche and migrant students with the rest of their classmates were usually carried out, also, on the basis of mutual respect. Among the aspects that favored such positive relationships should be mentioned that of the incorporation of TSG associated with Mapuche culture and countries of foreign students during the classes:

*The Mapuche-Huilliche children know better the “games” of their ethnic group, the other classmates approach them, and ask them for help to learn* (O-CH-34).

* My son says he likes it when the teacher plays games from our country (…) he teaches the other classmates how to play (…) the other children want to play with him* (DI-P-CH-02).

Another contributing factor may have been the introduction of cooperative activities, which fostered the coexistence of students and their acquisition of more social capital, especially during the meetings on Fridays, when students from other educational centers met. Here is one testimony in this regard:

*I like it when we go to other schools on Fridays, I met several guys with whom we sometimes get together to play soccer in the afternoons* (GI-MM-CH-05).

* Since we arrived in Chile, my son had had problems making friends, but since he started playing sports at school that changed, he got to know his classmates and kids from other schools better* (DI-P-CH-03).

The program continuously promoted social values and a positive classroom climate, however, isolated situations where negative social interactions developed among these students. Mapuche-Huilliche students and immigrant were sometimes discriminated against for cultural, racial, and socioeconomic reasons.

*A child tells the teacher that he does not want to play on the same team as two Mapuche-Huilliche students, expressly saying “those Indians are useless” in a dismissive tone* (O-CH-10).

* In one class, a Chilean boy says “I don’t want him on my team [a immigrant classmate], let him go to his country [he laughs mockingly], they are bad at playing ball [referring to football]”* (O-CH-09).

### 4.3. Interpersonal relationships between SCSP teachers and cultural minority students

This category deals with the relationships that SCSP teachers established with immigrant students in study one, and with Mapuche-Huilliche and immigrant students in study two. We show how these interactions either favor or hinder the process of social inclusion processes.

#### 4.3.1. Subcategory: Relationships between SCSP teachers and immigrant students in Spanish context

The relationships between teachers and immigrant students are different in each school. In this case study, a high level of teachers’ professional experience seems to be decisive in this sense, regardless of the number of immigrants that exists in their class. More novice teachers tended to have more problems with immigrant students: *“It is difficult for me to work with these [immigrant] children; they are undisciplined, and I do not know what to do with them”* (DI-T-SP-04).

Both the program coordinator and some teachers stated that they did not receive sufficient specific training to work with immigrant students. This is a worrying reality. It is not logical that the skills needed to manage adequate intercultural coexistence among the students may depend on whether teachers have acquired these on their own. This is evident to the program coordinator: “*Our teachers do not receive any training to work with immigrants, although the oldest ones have more experience in this regard*” (DI-C-SP).

The experienced SCSP teachers usually incorporated TSG linked to the countries of origin of their immigrant students and tended to be intolerant of situations of discrimination against immigrant students. “*I have experience working with foreign children. I am old in the programme. I use some games that [immigrant children] teach me*” (DI-T-SP-02). Foreign students valued these aspects positively.

#### 4.3.2. Subcategory: Relationships of SCSP teachers with Mapuche-Huilliche and immigrant students in Chilean context

The teachers of this SCSP program did not have any university training to work in cultural diversity contexts (even though one of the teachers had experience with foreign students), but received a basic training to work into the SCSP program. In this sense, the relations between these students and teachers were usually cordial. The students appreciated that the teachers show respect toward, and interest in, the Mapuche culture and traditions of other countries represented by immigrant students, in addition to a positive predisposition toward the learning of elements linked to it:

*My son told me that he likes to come to SCSP because now the teacher makes them play our [Mapuche-Huilliche] games* (DI-P-CH-01).

* At the beginning (first weeks) I was a bit bored, but now I like coming here (…) the teacher does things similar to what we used to do in my country (…) I haven’t played my favourite games for a long time* (GI-FM-CH-02).

This attitude toward the teachers was perceived as atypical and surprising (especially for Mapuche-Huilliche students), but favored positive relationships. In fact, during the implementation of typical games linked to the Mapuche people or immigrant children, teachers are open to receiving criticism and suggestions from students belonging to this cultural groups, generating a bidirectional learning space, where teachers and students learn from each other. Although the teacher’s previous experience proves to be an important factor in developing positive relationships.

*During class, a Mapuche-Huilliche student explains to the teacher how to play [a Mapuche game], the teacher listens attentively and thanks him* (O-CH-35).

* When my son started to go [to SCSP], he didn’t like the teacher [the younger teacher without previous experience with cultural minorities]. He said that sometimes the class was very chaotic* (…*). His older brother told me the same thing (…) but the other teacher [who did have previous experience in such contexts] was nice* (DI-P-CH-03).

### 4.4. Tensions generated by cultural stereotypes about the body and the practice of physical sporting activities

In both research scenarios, subjects, and groups with different worldviews and cultural practices converge. By virtue of this dynamic, there are situations where tensions or differences are observed around habits and beliefs linked to sports practice. This category presents these situations in both settings. First, we will discuss the cultural tensions experienced by immigrant students in the Spanish program, and then, later address the cultural tensions experienced by Mapuche-Huilliche and immigrant students in Chile.

#### 4.4.1. Subcategory: Tensions caused by cultural stereotypes in Spanish context

In the first case, immigrant students usually expressed a positive opinion about the SCSP program, although sometimes situations arose that contradict the codes and cultural worldviews of their places of origin. In concrete terms, it was observed that some immigrant girls (particularly from Latin America, Eastern Europe, and North Africa) refused to participate in some activities proposed by the teachers for considering those inappropriate for women: “*I cannot play that, they are games for men, if my mother or father saw me, they would be angry*” (GI-FI-SP-01). Curiously, some male students also considered that certain activities (e.g., football) were exclusively for males, resisting the fact that in the SCSP program these activities were organized for both genders. As described above, this revealed the existence of cultural gender stereotypes regarding physical expression and sports practice. It is important to mention that the teachers of each educational center promoted gender equality for all groups, natives and immigrants alike.

Also, there were immigrant students who rejected sports practices that were alien to their culture of origin. When they did not know any TSG or did not understand them, they preferred not to participate in those activities. This situation, as well as the resistance toward certain sports for reasons of gender, affected, at first, the levels of participation of immigrant students, hindering the social inclusion process: “*The teacher makes us play weird things, those sports are not played in my country, I do not like them*” (GI-MI-SP-08).

#### 4.4.2. Subcategory: Tensions caused by cultural stereotypes in Chilean context

In the second case study, as with the Spanish case, some tensions were caused by culture stereotypes in the classes and sport meetings around three dimensions that hindered social inclusion processes (because of the impact on interpersonal relationships and student participation).

First, there was a lack of interest on the part of Mapuche-Huilliche students to participate in activities that they deemed as alien to their context and culture. It was the same for some foreign students, who during the first weeks did not want to participate in games they had little mastery of.

*I do not like these sports [referring to modified rugby and hockey games]. We have never played them and do not know the rules* (…) *are from other countries* (GI-MM-CH-04).

* Today some children (coming from other countries) tell to teacher, they don’t want participate into the class games (…) they say that are “freak games” and boring* (…) *they never played them in their country* (O-CH-07).

Second, some immigrant and Mapuche-Huilliche students were reluctant to participate in some activities because of gender prejudices. That is, from their particular worldview, they considered certain bodily practices inappropriate for men or women. In short, they established what is culturally legitimate for each gender in terms of physical expression, as well as those behaviors that are improper and that can generate social disrepute if they are practiced (e.g., for them, it would be inappropriate for a girl to play football because they considered that to be unsuitable for females):

*There are girls who do not want to participate in some activities, they have complained that in some classes there are only activities for men* (DI-C-CH).

* My mother and grandmother tell me that some games are not for girls. That’s the way in my country* (GI-FI-CH-02).

Third, some Mapuche-Huilliche students openly stated that school is not a priority for them. For them, the most relevant educational process is one that is built within the indigenous communities themselves, where alternative types of knowledges are promoted. Some Mapuche-Huilliche students considered that many contents taught in school, including some of the SCSP program, insignificant to their lives.

*Mapuche-Huilliche students sometimes tell me that at school they do not learn anything important, for them and their families it is more important what they learn in their culture (…), that is why sometimes they do not come to school* (DI-T-CH-01).

## 5. Discussion

Regarding the interpersonal relationships between students of cultural minorities groups, the results show that participation in these SCSP programs, for the most part, had positive effects in terms of social inclusion in both contexts. Although in both settings existed conflicts, especially stemming from the prejudices of natives and the newcomers, who were not yet familiarized with the SCSP principles and dynamics, the interpersonal relationships of this students could be considered as positive, especially considering the collaborative solidarity practices and empathy demonstrated during the classes and meetings. This may be due to the use of learning activities, focused on the development of social cohesion, cooperation, and non-competitiveness; as well as the introduction of TSG belonging to the cultures of the minority groups and the sport motor competence that students display. Previous research shows that such practices not only are recognized as strengthening symbols of cultural identity for immigrant and native ethnicities, but also promote harmonious social relationships among individuals from different origins and traditions even in communities without immigrants or indigenous peoples (Román, 2010; Carter-Thuillier et al., 2018; Berti and Lapiccirella, 2019; Puente-Maxera et al., 2020). In short, TSG are cultural traditions and symbols (Martín-Martínez et al., 2021) that allow, through physical-playful experiences, the conservation of identities and the positive exchange between them based on corporeality (Parlebas, 2020). Therefore the use of TSG in the classroom is a concrete way of recognizing the different cultures present in the context, assuming then, that human action and motricity are indivisibly linked with culture (Martínez-Santos et al., 2020).

In both contexts, students are fluent speakers of Spanish, enabling communication between them. However, Spanish is spoken because institutional contexts force students to speak it, the official language, and Mapuche students are discriminated against if they speak their traditional language. Therefore, students have no choice. The above, in addition to the systematic exclusion and pauperization of Mapuche culture into the Chilean schools (Turra et al., 2017; Nahuelpan and Antimil, 2019; Poblete et al., 2020), may be affecting that students belonging to the Mapuche-Huilliche ethnic group show a progressive loss of their ancestral language and partial ignorance of their TSG is a worrying sign. In the same direction, the literature suggests that negative acculturation processes sometimes take place from the dominant culture (Berry, 2017), particularly when subordinate relations are established with minority groups, affecting the development and conservation of their cultures (Banks, 2016); this could explain the weakening of the Mapuche-Huilliche culture and its ancestral language (Williamson et al., 2012). For this reason, it is positive that SCSP programs examined in this study used TSG linked with all cultural identities in classes and meetings, since different authors (Dubnewick et al., 2018; Berti and Lapiccirella, 2019; Luchoro-Parrilla et al., 2021; Saura and Zimmermann, 2021) have pointed out the need to promote the development of cultural identities through immaterial aspects such as TSG. Moreover, it is a learning opportunity for all students and positively valued by both native and cultural minority students.

In terms of interpersonal relationships between cultural minorities students and other children, the results show that both SCSP programs promoted positive relationships by facilitating the development of new social networks among the members of different cultures, which otherwise would not exist. This is consistent with other previous studies conclusions (Verhagen and Boonstra, 2014; Smith et al., 2019; Flensner et al., 2021; Ekholm et al., 2022), stating that sports activities among subjects of different cultural and ethnic origin encourage the acquisition of social capital for all individuals, promoting the cultural capital (re)production of identities into the context, and then allowing the processes of social inclusion (Spaaij, 2012; Mohammadi, 2022). In this vein, other researchers have suggested that sports can be a privileged medium for the development of intercultural competence in children students (Grimminger-Seidensticker and Möhwald, 2017). Needless to say, these researchers’ conclusions ought not to be admitted at face value. There are certain limitations, which, in fact, were obvious in both SCSP programs, where conflicts did emerge and which were directly related to ethnocentric ideologies, discourses and practices (Banks, 2016), in addition, according to the study findings, children who have low sport motor competencies may face somewhat more complex processes in this respect. However, the incorporation of TSG linked to the cultures present in each context proved to be an effective strategy to favor rapprochement and relations between students. According to Saura and Zimmermann (2021), this can be explained because incorporate TSG of all identities is synonymous to recognize each culture and members of them, also an opportunity to promote rapprochement, dialogue, learning, and socially inclusive climates. However, it is always important to critically analyze these inclusive alleges properties of sport, as in many cases specific skills are required to take part in them, as well TSG are gender-stereotyped also.

As for the interpersonal relationships between SCSP teachers and cultural minorities students, these case studies data indicate that, in both contexts, educators would have benefited from specific training to work in culturally diverse contexts. This lack of training can be made up with prolonged experience in pluri-cultural contexts; although, for those who do not have it, it is very difficult to prevent and solve conflicts, especially those related to intercultural and inter-ethnic situations (Flores et al., 2014; Wyant et al., 2020). Furthermore, this shortfall has negative effects on students, regardless of their cultural or ethnic background. This was obvious, especially, in the case study carried out Spain. In relation to the above, different authors (Grimminger, 2011; Ko et al., 2015; Wyant et al., 2019, 2020; Doidge et al., 2020; Siljamäki and Anttila, 2021) have raised the need to include intercultural competence in the training of PE teachers and Sport Coaches, because otherwise they might have initial problems in working in culturally diverse contexts and also difficulties in helping their students to acquire intercultural skills. Likewise, studies (Carter-Thuillier et al., 2018; Pic et al., 2019; Puente-Maxera et al., 2020; Martín-Martínez et al., 2021; Lavega-Burgués et al., 2023; Moya-Higueras et al., 2023) related to TSG show that its use in the classroom represents an effective way of incorporating different cultures into the school, in addition to generating positive effects on different socioemotional and motor variables, however, the need for teachers who are sufficiently prepared in this area is also exposed for authors. Therefore, it seems essential to specifically train teachers who intend to work with the use of TSG in intercultural contexts.

It became evident that participation in sport activities at school context not resolve full tensions about diverse cultural ideologies, discourses and traditions, it can sometimes even aggravate them if intercultural approaches are not used. The mere application of foreign cultural ideals about the body and movement can generate anthropological, sociological, gender-related, and theological tensions (Lenneis and Pfister, 2017; Thorpe et al., 2022). Some of this was evident at the beginning of SCSP programs. To prevent this, a specific intercultural pedagogy is essential to compliment the possibilities for intercultural understanding and cooperation that both schooling and sport offer. In this sense, different authors (Shea and Beausoleil, 2012; McSweeney and Hakiza, 2022; Thorpe et al., 2022; Truskewycz et al., 2022) raise the need to analyze how the body representations of immigrant and native groups are limited by dominant cultural traditions and institutions, for this reason overcoming cultural stereotypes in relation to sport and movement, especially those that segregate girls, requires a deep understanding of these cultural systems in order to succeed in this task, otherwise resistance from members of these cultures is likely to be encountered.

## 6. Conclusion

Both programs demonstrate favor social inclusion processes in culturally diverse contexts, facilitating the creation and development of interpersonal relationships between students with different cultural identities, interactions that are mostly based on mutual respect and support. However, there are also conflictive situations, especially during the initial commencement of each program (perhaps because the students were not yet adapted to the methodological proposal of the programs), as well as prejudice and reciprocal discrimination between children who belong to cultural minorities and those who do not in both contexts. In addition, the sport motor competence level of each student seems to have an impact on socialization processes, who are less proficient experience more complex processes in this regard.

The incorporation of TSG belonging to all the cultures present in the context, as well as the experience and level of preparation of the teachers to work in intercultural educational environments, seem to be determining aspects to favor the processes of social inclusion. However, considering the findings of this study, the stereotypes that each culture has about sport and movement also prove to be a determining aspect to consider, especially when become a reason for exclusion or segregation (as sometimes happens with girls in both cases). However, it is also essential to look closely at TSG and their gender-stereotyped, because they can exacerbate conflicts and segregation situations.

The findings of this multi-case study are only valid for the contexts where the research was carried out. However, although the reality of cultural minorities is subject to the particularities of each social space, the results of this research indicate that in both contexts there are similar aspects that favor and hinder the processes of social inclusion, and also that after-school SCSP programs can enhance them when proposals focused on the development of socio-educational values are developed.

In closing, constructing a specific pedagogy based on these findings might be useful to teachers and sports coaches who work in contexts of cultural diversity, especially in scenarios with pluri-ethnic and multi-national students. This research can also be suitable to professionals in the Social Sciences, whose interest is in the education and social relations of immigrant and native ethnic groups.

## Data availability statement

The raw data supporting the conclusions of this article will be made available by the authors, without undue reservation.

## Ethics statement

The studies involving human participants were reviewed and approved by the Academic Committee of the Doctoral Program in Transdisciplinary Research in Education at University of Valladolid (Spain) and the Bioethics and Biosafety Committee of the Universidad de Los Lagos (Chile). Written informed consent to participate in this study was provided by the participants and legal guardians as appropriate in each case.

## Author contributions

BC-T, VL-P, and FG-F defined the design, participated in the data collection, and wrote the manuscript. JC-B and J-MF-B participated reviewing the methodological design and collaborated to edit and write the manuscript. PD-F, EG-S, and AS participated on the manuscript writing and contributed to the final data analysis. All authors contributed to the article and approved the submitted version.
